# Workload, Harassment, and Workplace Consultation Availability and Their Associations with Mental Distress among Physical Therapists

**DOI:** 10.1298/ptr.25-E10390

**Published:** 2026-04-29

**Authors:** Munetsugu KOTA, Sae UEZONO, Toru NISHIGORI, Tetsuya CHIBA, Kiyoshi NAWAI

**Affiliations:** 1 Department of Rehabilitation, Hiroshima Cosmopolitan University, Japan; 2 Japanese Society of Physical Therapy in Mental and Psychological Domains, Japan; 3 Department of Education, Ageo Central Medical College, Japan; 4 Department of Rehabilitation, Ohata Hospital, Japan; 5 R Professional University of Rehabilitation, Japan

**Keywords:** Mental distress, Overtime, Workplace harassment, Workplace consultation availability, Physical therapists

## Abstract

**Objectives:**

Physical therapists (PTs) face various psychosocial risks that affect their mental health; however, profession-specific reports on organizational exposures remain limited. This study aimed to examine total overtime, workplace harassment, and workplace consultation availability and their associations with current mental distress among PTs in Japan.

**Methods:**

A nationwide cross-sectional web survey of PTs, conducted from August 12 to September 20, 2025, was analyzed. The primary outcome was current mental distress (yes/no). The main exposures were total overtime in the past 3 months (per 10 h/3 months), workplace harassment (yes/no), and workplace consultation availability (yes/no). Separate multivariable logistic models were fit for each exposure with a common adjustment set (sex, age, tenure in current workplace [midpoint, years], employment status, hospital bed size, and number of PTs). Missing covariates were handled via multiple imputation (multiple imputation by chained equations, m = 15) with pooling by Rubin’s rules.

**Results:**

Of the 718 PTs surveyed, 312 (43.5%) were classified as distress present. Mean total overtime was 16.7 ± 15.0 h over 3 months and was higher in the distress-present group. Adjusted associations were total overtime adjusted odds ratio (aOR) 1.13 (95% confidence interval [CI] 0.98–1.31) per additional 10 h, harassment aOR 2.29 (95% CI 1.67–3.14), and workplace consultation availability aOR 0.60 (95% CI 0.43–0.84).

**Conclusions:**

Workplace harassment was associated with higher rates of mental distress, whereas workplace consultation availability was inversely associated. Total overtime showed a positive trend in the adjusted model. These findings point to organizational targets, including overtime management, harassment prevention, and accessible consultation routes.

## Introduction

Physical therapists (PTs) play a central role in rehabilitation contexts, yet they face numerous psychosocial demands, including heavy caseloads, emotionally taxing encounters with patients, and complex interprofessional coordination, that may elevate the risk of poor mental health outcomes and burnout.

Profession-specific reviews indicate a significant burden of burnout among PTs, with organizational and workload factors frequently implicated^1)^. Evidence from allied health and nursing further underscores implications for care quality, linking clinician burnout to poorer safety climate and patient experience^2)^. Broader clinical research has also reported a substantial depressive symptom burden in training-intensive roles^3)^.

Among the modifiable workplace exposures, long working hours and overtime have been repeatedly associated with depressive symptoms and mental distress^4,5)^. Workplace harassment and violence are likewise linked with anxiety, depression, and burnout in healthcare settings^6,7)^. In contrast, access to supportive resources such as psychological safety, effective teamwork climates, and clear consultation channels aligns with stress-buffering theory and is associated with lower burnout and better mental health outcomes^8–11)^. In Japan, policy attention to excessive overtime has led to statutory caps and monitoring, underscoring the salience of overtime as a modifiable risk^12)^.

This study aimed to evaluate the associations of total overtime, workplace harassment, and workplace consultation availability with current mental distress among PTs in Japan, using multivariable models adjusted for demographic and facility characteristics. As descriptive context, related indicators were summarized (e.g., leave due to distress and consultation channels).

## Methods

### Study design and participants

A cross-sectional, web-based survey of PTs in Japan was conducted from August 12 to September 20, 2025, and the data were analyzed. Multiple academic societies within the Japanese Society of Physical Therapy—the Japanese Society of Physical Therapy for Policies and Administration, the Japanese Society of Physical Therapy in Mental Health, the Japanese Society of Physical Therapy for Oncology and Lymphedema, and the Japanese Society of Physiotherapy in Occupational Health—distributed the survey link via member e-mails and notices on their websites. Participation was voluntary and anonymous; consent was indicated by selecting the consent button on the landing page. All 718 responses were eligible because the primary outcome and main exposures had no missing values. No direct identifiers were collected. This study was approved by the Ageo Central Medical Vocational School Ethics Committee (approval number: 25-0002).

### Measures

Outcome: Current mental distress was assessed using a binary self-report (yes/no to experiencing stress, severe worry, or anxiety at present) and served as the dependent variable.

Main exposures: Total overtime in the past 3 months was captured as the sum of overtime hours. For analysis, we interpreted effects per additional 10 h over 3 months and mean-centered the variable (mean = 16.7 h/3 months) to aid interpretation. Workplace harassment was captured using a binary indicator (yes/no). For descriptive purposes, respondents who reported workplace harassment were also asked about harassment types (power, moral, sexual, and other); each type was coded as a binary indicator, and “any harassment” was defined as reporting at least 1 type. Consultation availability was captured using a single binary item that asked whether a consultation source was available within the workplace.

Covariates: An adjustment set was pre-specified: sex (female = 1), age (midpoint of band, years), tenure in current workplace (midpoint, years), employment status (full-time = 1), hospital bed size (per 100 beds), and number of PTs (midpoint of band).

### Missing data

Binary outcomes and main exposures had no missing values. The remaining missing covariates were addressed using multiple imputation by chained equations (m = 15) and pooled with Rubin’s rules^13,14)^.

### Statistical analysis

For 2-group comparisons in Tables 1 and 2, we used the Mann–Whitney U test for continuous variables, Fisher’s exact test for binary variables (with chi-squared approximation when expected counts were adequate), and chi-squared tests of independence for multi-level categorical variables. For multi-response items (e.g., harassment types, consultation channels), each level was summarized with the appropriate denominator (experience-restricted or valid responses) and compared descriptively; corresponding p-values for the overall distribution were reported where applicable.

**Table 1.** Participant and facility characteristics (overall and by current distress status)

Level	Total (n = 718)	Distress present (n = 312)	Distress absent (n = 406)	p-Value
Sex, n (%)				0.358
Female	215 (29.9)	97 (31.1)	118 (29.1)	
Male	499 (69.5)	212 (67.9)	287 (70.7)	
Other/unspecified	4 (0.6)	3 (1.0)	1 (0.2)	
Age, n (%)				0.792
20s	145 (20.2)	57 (18.3)	88 (21.7)	
30s	184 (25.6)	79 (25.3)	105 (25.9)	
40s	245 (34.1)	109 (34.9)	136 (33.5)	
50s	116 (16.2)	54 (17.3)	62 (15.3)	
60s	28 (3.9)	13 (4.2)	15 (3.7)	
Tenure in current workplace, n (%)				0.767
<1 year	76 (10.6)	28 (9.0)	48 (11.8)	
1–3 years	119 (16.6)	55 (17.6)	64 (15.8)	
4–10 years	200 (27.9)	86 (27.6)	114 (28.1)	
10–<20 years	196 (27.3)	87 (27.9)	109 (26.8)	
≥20 years	127 (17.7)	56 (17.9)	71 (17.5)	
Position rank, n (%)				0.990
No position	357 (49.7)	152 (48.7)	205 (50.5)	
Unit lead	12 (1.7)	6 (1.9)	6 (1.5)	
Section chief	136 (18.9)	57 (18.3)	79 (19.5)	
Manager	91 (12.7)	41 (13.1)	50 (12.3)	
Department head	107 (14.9)	49 (15.7)	58 (14.3)	
Other position	13 (1.8)	6 (1.9)	7 (1.7)	
Other/unspecified	2 (0.3)	1 (0.3)	1 (0.2)	
Employment status, n (%)				0.168
Full-time	691 (96.2)	304 (97.4)	387 (95.3)	
Part-time	27 (3.8)	8 (2.6)	19 (4.7)	
Working hours (h/day)	7.9 ± 1.4 (n = 718)	7.9 ± 1.0 (n = 312)	8.0 ± 1.6 (n = 406)	0.365
Paid leave usage (%)	62.3 ± 73.2 (n = 712)	59.4 ± 31.3 (n = 310)	64.5 ± 93.4 (n = 402)	0.656
Hospital bed size (bed)	219.1 ± 235.0 (715)	209.1 ± 211.9 (311)	226.7 ± 251.4 (404)	0.921
Number of physical therapists, n (%)				0.503
<5	100 (13.9)	45 (14.4)	55 (13.5)	
5–<10	94 (13.1)	38 (12.2)	56 (13.8)	
10–<20	162 (22.6)	78 (25.0)	84 (20.7)	
≥20	362 (50.4)	151 (48.4)	211 (52.0)	
Number of rehabilitation professionals, n (%)				0.778
<10	131 (18.2)	54 (17.3)	77 (19.0)	
10–<20	106 (14.8)	48 (15.4)	58 (14.3)	
20–<50	212 (29.5)	97 (31.1)	115 (28.3)	
≥50	269 (37.5)	113 (36.2)	156 (38.4)	
Facility function, n (%)				0.251
Medical institution	565 (78.7)	255 (81.7)	310 (76.4)	
Long-term care facility/agency	69 (9.6)	26 (8.3)	43 (10.6)	
Educational/research institution	40 (5.6)	12 (3.8)	28 (6.9)	
Company	11 (1.5)	5 (1.6)	6 (1.5)	
Government/agency (non-healthcare)	9 (1.3)	2 (0.6)	7 (1.7)	
Other (none of the above)	24 (3.3)	12 (3.8)	12 (3.0)	

Working hours: Contracted working hours (h/day). Paid leave usage: Rate of paid annual leave taken in the previous fiscal year. Participant and facility characteristics are shown for the distress-present and distress-absent groups. Values are n (%) unless noted; continuous variables are mean ± standard deviation (SD) (n). Two-group p-values are displayed in the rightmost column.

**Table 2.** Prevalence of mental health indicators and key exposures (overall and by current distress status)

	Total (n = 718)	Distress present (n = 312)	Distress absent (n = 406)	p-Value
Mental health indicators				
History of mental distress, n (%)	512 (71.3)	293 (93.9)	219 (53.9)	<0.001
Received medical care^a^, n (%)	103 (14.3)	61 (19.6)	42 (10.3)	<0.001
Medication use for mental distress, n (%)	96 (13.4)	62 (19.9)	34 (8.4)	<0.001
Leave taken due to mental distress, n (%)	90 (12.5)	57 (18.3)	33 (8.1)	<0.001
Leave duration^b^, n (%)				
1 day	25 (27.8)	18 (31.6)	7 (21.2)	0.396
2–6 days	29 (32.2)	15 (26.3)	14 (42.4)	
≥1 week	8 (8.9)	6 (10.5)	2 (6.1)	
≥1 month	28 (31.1)	18 (31.6)	10 (30.3)	
Workload and employment conditions				
Scheduled working hours (h/day)	7.9 ± 1.4 (718)	7.9 ± 1.0 (312)	8.0 ± 1.6 (406)	0.365
Paid leave uptake in prior year (%)	59.8 ± 31.7	59.2 ± 31.2	60.2 ± 32.2	0.677
Overtime pay provided (contract), n (%)	563 (78.4)	248 (79.5)	315 (77.6)	0.583
Total overtime in past 3 months (h)	16.7 ± 15.0 (151)	19.5 ± 14.8 (63)	14.8 ± 15.0 (88)	0.035
Unreported overtime ratio				
0	227 (31.6)	77 (24.7)	150 (36.9)	<0.001
>0	333 (46.4)	170 (54.5)	163 (40.1)	
Unknown	158 (22.0)	65 (20.8)	93 (22.9)	
Harassment				
Workplace harassment experience, n (%)	348 (48.5)	186 (59.6)	162 (39.9)	<0.001
Type of harassment, n (%)				
Power harassment	289 (83.0)	156 (83.9)	133 (82.1)	0.670
Moral harassment	145 (41.7)	81 (43.5)	64 (39.5)	
Sexual harassment	34 (9.8)	18 (9.7)	16 (9.9)	
Other	20 (5.7)	13 (7.0)	7 (4.3)	
Consultation system				
Workplace consultation availability, n (%)	513 (71.4)	206 (66.0)	307 (75.6)	0.006
Designated contact person^c^, n (%)	406 (56.5)	166 (53.2)	240 (59.1)	0.129
Type of consultation channel, n (%)				
Formal/professional	207 (40.4)	89 (43.2)	118 (38.4)	0.313
Management line	271 (52.8)	88 (42.7)	183 (59.6)	
Informal/peer	115 (22.4)	56 (27.2)	59 (19.2)	

Prevalence estimates are shown for the distress-present and distress-absent groups. Values are n (%) unless noted; continuous variables are mean ± standard deviation (SD) (n). Percentages for harassment type/impact are among those who reported harassment; percentages for consultation channels use valid respondents. Definitions and additional details are provided in the Methods section.

^a^Received medical care for mental distress.

^b^Leave duration among those who took leave.

^c^Designated contact person for consultation.

We fit 3 multivariable logistic regression models corresponding to the main exposures: (i) labor time model (total overtime, per 10 h/3 months, mean-centered), (ii) harassment model (any workplace harassment vs. none), and (iii) workplace consultation model (workplace consultation availability vs. none). We also fit type-specific models in which the “any workplace harassment” exposure was replaced with each harassment-type indicator separately (power, moral, sexual, and other), using the same covariate set and pooling approach. Adjusted odds ratios (aORs) with 95% confidence intervals (CIs) were calculated. Sensitivity analyses exploring nonlinearity and alternative consultation definitions informed development but were not emphasized in the final reporting. Two-sided p <0.05 was considered statistically significant. Analyses were conducted in Python 3.11.8 (pandas 1.5.3; NumPy 1.24.0; statsmodels 0.13.5; scikit-learn 1.1.3; matplotlib 3.6.3; openpyxl 3.0.10) and R 4.5.1.

## Results

Of the 718 PTs participating, 312 (43.5%) were classified as distress present. Table 1 summarizes demographic and facility characteristics overall and by distress status. Between-group tests did not indicate statistically significant differences across key attributes.

Table 2 presents mental health indicators (e.g., prior distress, medical care and medication for distress, and leave due to distress and its duration) and workload/employment conditions (e.g., scheduled hours, paid leave uptake, overtime pay, total overtime, and unreported overtime ratio), as well as harassment-related items and consultation channels. In type-specific models, power harassment (aOR 2.07, 95% CI 1.51–2.83) and moral harassment (aOR 1.86, 95% CI 1.28–2.71) were associated with current mental distress, whereas sexual harassment showed no clear association.

In adjusted models (Table 3 and Fig. 1), total overtime showed a positive association with distress: aOR 1.13 (95% CI 0.98–1.31) per additional 10 h over 3 months. Any workplace harassment was associated with higher rates of distress: aOR 2.29 (95% CI 1.67–3.14). Workplace consultation availability was associated with lower rates of distress: aOR 0.60 (95% CI 0.43–0.84).

**Table 3.** Multivariable logistic regression (MI pooled): main-effect models

Model	Predictor	aOR	95% CI
Labor time model	Total overtime (per 10 h/3 monts)	1.13	0.98–1.31
Harassment model	Harassment experience (yes vs. no)	2.29	1.67–3.14
Consultation model	Workplace consultation availability (yes vs. no)	0.6	0.43–0.84

aORs with 95% CIs are shown for 3 models: labor time (total overtime, per additional 10 h/3 months), harassment (any vs. none), and consultation availability (yes vs. no). Model specification and adjustment details are described in the Methods section.

aOR, adjusted odds ratio; CI, confidence interval; MI, multiple imputation

**Fig. 1.**
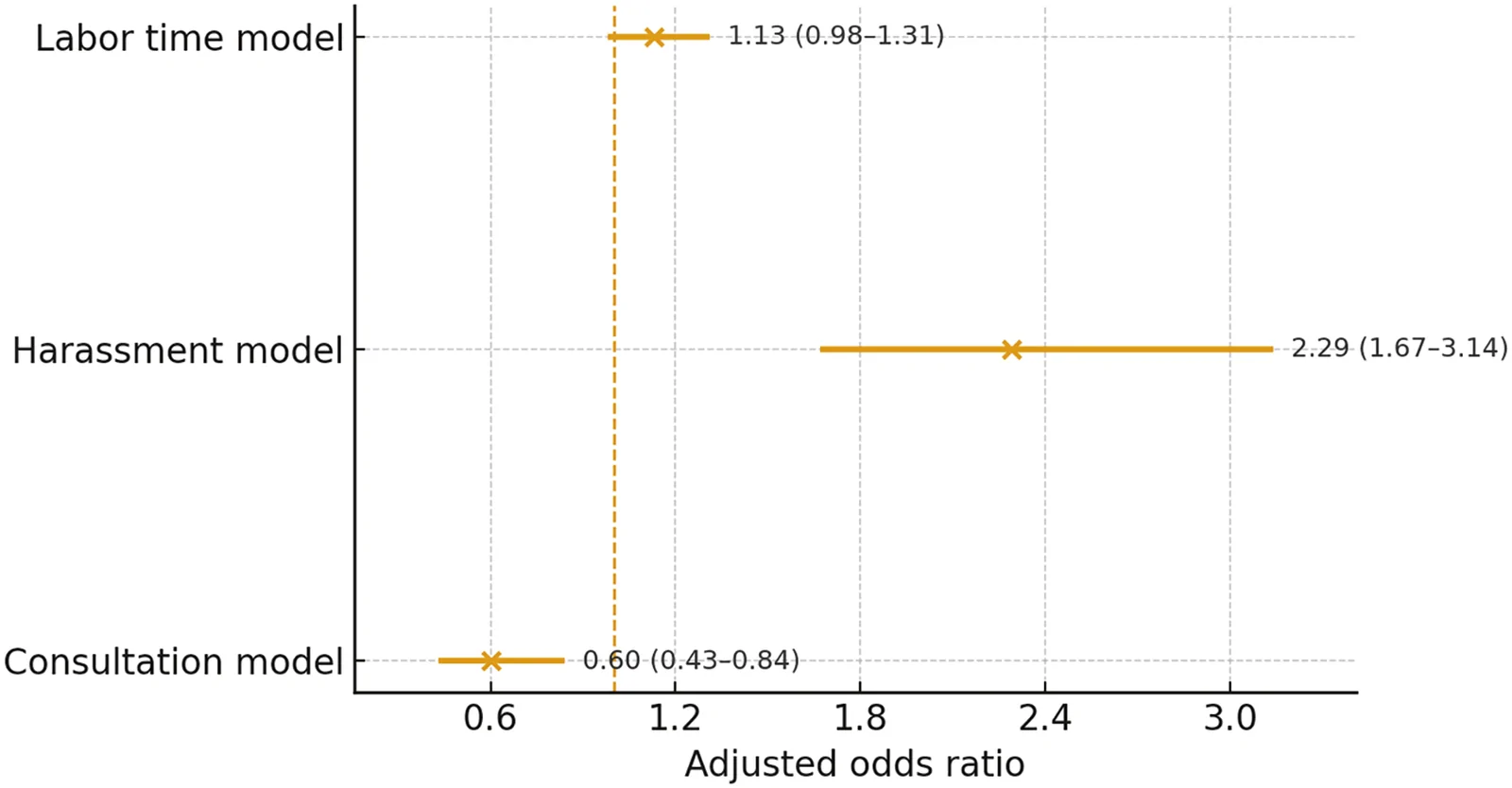
Adjusted odds of current mental distress by workplace factors This forest plot presents aOR (95% CIs) from 3 separate models: the labor time model (total overtime, per additional 10 h over the past 3 months), the consultation model (workplace consultation availability, yes vs. no), and the harassment model (any vs. none). The horizontal axis indicates aOR = 1. aOR, adjusted odds ratio; CI, confidence interval

## Discussion

In a national sample of Japanese PTs, workplace harassment was a salient correlate of current mental distress, whereas workplace consultation availability showed a protective association; total overtime showed a positive, but nonsignificant, trend after adjustment. These associations persisted after adjusting for demographic and workplace characteristics and multiple imputation. Collectively, the results highlight workload and mistreatment as modifiable risks and consultation availability as a plausible organizational buffer.

The trend of higher distress with greater overtime accords with broader clinical literature documenting depressive symptoms and burnout owing to heavy work hours and training demands^3–5)^. In Japan, labor policy reforms imposing overtime caps underscore the recognition of overtime as a modifiable health risk^12)^. The study expressed overtime effects per additional 10 h/3 months, a unit that is straightforward to interpret and broadly consistent with policy-relevant ranges. Even when average overtime appears moderate, patient-facing and psychologically demanding tasks can make relatively small amounts consequential. Unreported overtime (>0) was more common among respondents with current distress than among those without distress (54.5% vs. 40.1%). This pattern may indicate a work climate where extra work is difficult to report or recognize. Such a climate could be linked to psychological burden through perceived unfairness, reduced control, and distrust toward the organization.

The strong association between harassment experience and distress mirrors evidence that bullying, harassment, and workplace violence are linked to worse mental health outcomes^6,7)^. In this survey, the specific contexts in which respondents perceived harassment were not ascertained (e.g., severity, repetition, power gradient, or location); therefore, future research should delineate these features to inform prevention measures focused on severe harassment that contributes to mental distress.

The inverse association is consistent with stress-buffering theory^11)^ and with organizational evidence linking psychological safety and supportive climates with lower burnout and better mental health outcomes among health workers^8–10)^. In practice, workplace consultation availability can be viewed as a multichannel structure complementing management-line controls on time and harassment: management-line routes enable early action and transparent follow-through, while formal/professional services and informal/peer support reduce barriers to help-seeking.

Study strengths include national scope, profession-specific focus, and a pre-specified analytic plan (multiple imputation with pooled multivariable models and harmonized codings). Limitations include cross-sectional and self-report design (no temporality; susceptibility to reporting biases for harassment and overtime, including unreported overtime) and possible residual confounding. Reverse causation is possible (e.g., distress may reduce work efficiency and increase overtime). Invitations were distributed via society e-mails and web notices; the true sampling denominator and response rate are unknown, raising the possibility of self-selection and nonresponse bias (e.g., greater participation among those with a strong interest in or experience of mental distress). Harassment was assessed by self-report; frequency, duration, and severity were not captured. A history of mental distress is likely multifactorial and may involve contributors beyond the focal workplace factors (e.g., interpersonal conflicts or family responsibilities), which were not fully assessed in this survey. The consultation measure was a binary availability indicator; future research should disaggregate formality, ease of access, perceived trust, and climate. Generalizability beyond the Japanese PT context requires caution.

## Conclusions

Among Japanese PTs, workplace harassment was associated with higher rates of current mental distress, while consultation availability was inversely associated, and total overtime showed a positive trend. Organizational approaches that combine hazard control with accessible consultation routes warrant evaluation in future intervention studies.
